# Nasal Sculpting: Calculated and Predictable Tip Elevation With Cephalic Trim

**Published:** 2015-06-01

**Authors:** Jeremiah S. Redstone, Saeed Chowdhry, Jonathan Nguyen, Durham Alan North, Ron Hazani, Brad Drury, Eric M. Yoder, Ross D. Cooperman, Virginia Yoder, Jarrod A. Little, Larry D. Florman, Bradon J. Wilhelmi

**Affiliations:** ^a^Department of Surgery, University of Louisville, Louisville, Ky; ^b^Department of Surgery, University of Illinois at Mt Sinai, Chicago

**Keywords:** nasal sculpting rhinoplasty, cephalic alar trim, nasolabial angles, lower lateral cartilage, nasal tip elevation

## Abstract

**Background:** Rhinoplasty techniques to affect nasal tip rotation are well described. Cephalic alar trim is a powerful method for achieving tip elevation. Previous studies and texts provide aesthetic guidelines for nasolabial angles. Often, surgeon experience determines the degree of lower lateral cartilage resection to achieve optimal results. This study analyzes the change in tip elevation with measured resections of the lower lateral cartilages. This can aid the surgeon in accurately predicting the effect of cephalic alar trim on tip elevation. **Methods:** Ten fresh cadaveric dissections were performed to determine the change in nasolabial angles after cephalic trim of the lower lateral cartilage. Closed rhinoplasty technique was performed using marginal and intercartilaginous incisions to expose the lower lateral cartilage. Caliper measurements of the lower lateral cartilage were recorded. Serial cephalic trim was performed in 25% increments. True lateral photographs were obtained before and after each serial excision. Nasolabial angle measurements were obtained using a digital goniometer for digital photo analysis. **Results:** Four female and 6 male cadavers were evaluated. The mean initial nasolabial angle was 106° ± 2°. The mean lower lateral cartilage width was 9.45 ± 1.38 mm. Serial 25% reductions in lower lateral cartilage height resulted in a mean total nasolabial angle change of 7.4°, 12.9°, and 19.6°, respectively. The mean incremental change in the nasolabial angle was 6.47° ± 1.25°. **Conclusion:** The nasolabial angle is an essential aesthetic feature. Cephalic trim is a key maneuver in affecting the nasolabial angle. A 25% lower lateral cartilage cephalic trim correlates with an average change in the nasolabial angle of 6.47°. Knowledge of the cephalic trim to nasolabial angle relationship aids in achieving desired tip elevation.

Rhinoplasty techniques to affect nasal tip rotation are well described. Cephalic alar trim is a powerful method for achieving tip elevation. Previous studies and texts provide aesthetic guidelines for nasolabial angles (NLAs). Often, surgeon experience determines the degree of lower lateral cartilage (LLC) resection to achieve optimal results. This study analyzes the change in tip elevation with measured resections of the LLC. This can aid the surgeon in accurately predicting the effect of cephalic alar trim on tip elevation.

## METHODS

Ten fresh cadaveric dissections were performed to determine the change in NLAs after cephalic trim. Closed rhinoplasty technique was performed using marginal and intercartilaginous incisions to expose the LLC. Caliper measurements of the LLC were recorded. Caliper measurements were again marked at 25% increments of the LLC. Twenty-five percent incremental cephalic trim was performed with true lateral photographs obtained before and after each serial excision. Predissection and postdissection photographs were taken with a 110-mm lens. From a lateral view, with the camera at the same elevation for all specimens, the viewfinder was focused on the Frankfort line at the lateral canthus at a fixed distance of 1.0 m. Focus was achieved by moving the camera and not by adjusting the lens.^1^ Nasolabial angle measurements were obtained using a digital goniometer with Adobe Photoshop (version 10.0; Adobe Systems Inc, San Jose, Calif) for digital photo analysis. An independent researcher not involved in the dissections performed these measurements randomly.

## RESULTS

**Figures 1a–d:**
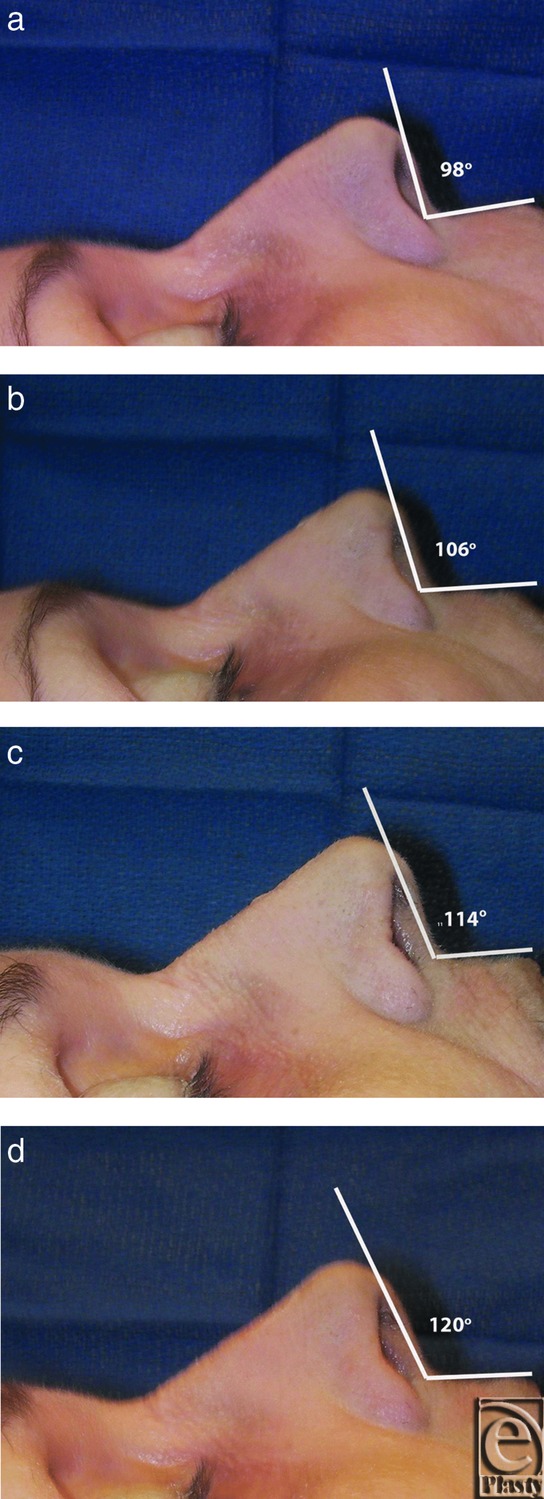
Fresh tissue dissections of specimen 8. Serial 25% cephalic alar trim and the corresponding nasolabial angle change are demonstrated.

Four female and 6 male cadavers were evaluated. The mean initial NLA was 106° ± 2°. The mean LLC width was 9.45 ± 1.38 mm. Serial 25% reductions in LLC height resulted in a mean total NLA change of 7.4°, 12.9°, and 19.6°, respectively. The mean incremental change in NLA was 6.47° ± 1.96°. See Figures 1a–1d and Tables 1 and 2.

## DISCUSSION

Rhinoplasty challenges many surgeons for a variety of reasons. Tremendous variation exists in the individual patient's nasal anatomy as well as in facial metric analysis and opinion on operative technique. Mastery of nasal anatomy and analysis of complete facial elements are essential to achieving pleasing outcomes in rhinoplasty. Surgeons must also possess in their arsenal of rhinoplasty techniques several maneuvers to effectively attack complex nasal surgery.

### Anatomy

A complete presentation of all the nasal elements is beyond the scope of this article. We focus primarily on the tip structures as they relate to tip position and rotation.

The medial, middle, and lateral crura and accessory cartilages make up the LLC and form scaffolding for the nasal tip. Fibrous attachments connect these elements to each other, the piriform aperture, and to the upper lateral cartilages. These fibrous attachments in conjunction with the lower and upper lateral cartilages, piriform aperture, and caudal septum form the basis for tip position.^2^^,^^3^ In addition to the skin and bony structures, the nasalis muscles and the depressor septi nasi muscles can also contribute to tip animation and position.^4^ The size and shape of the LLC may also produce nasal tip asymmetries.^5^ The strength and attachments of the LLC also have been shown to impact the position of the nasal tip.^6^^-^8 Alterations in these elements will adjust the position of the tip.

**Figure 2:**
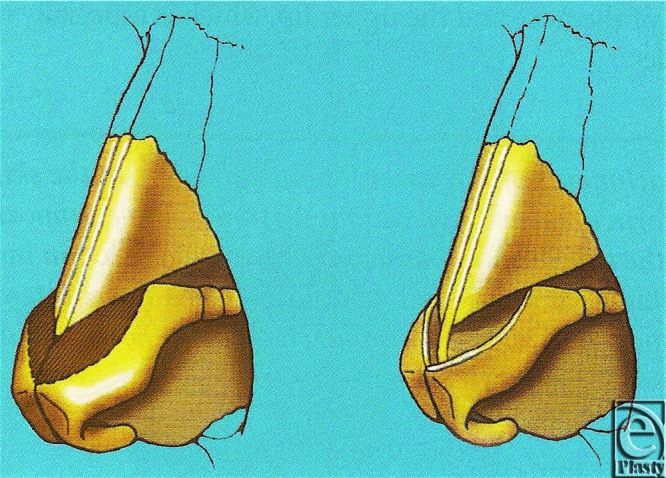
Illustration of the cephalic trim technique altering tip position. Reprinted from Gunter and Hackney.^2^

The “tripod concept” simplifies these anatomical elements. The nasal tip can be described as a tripod of 3 cartilaginous legs. With the patient upright, the tripod is formed with 1 inferior leg and 2 superior legs; the lower leg is formed by the medial crura, and the lateral crural complexes form the upper legs with attachments to the piriform aperture (Fig 2).^2^^,^^6^^,^^7^^,^^9^^-^12 As a result, adjusting the length of any of these structures will alter the position of the tip.

### Facial metrics

Preoperative planning in rhinoplasty is essential for optimizing outcomes. The surgeon needs to assess all the facial elements when planning rhinoplasty. The goal of rhinoplasty is to create harmony with all the elements of the face.^13^ Several measurements have been developed using anatomical landmarks to identify ideal facial aesthetics. Some suggest that the face can be divided into 3 equal portions, with the distance from nasion to nasal base equaling the distance from hairline to nasion and nasal base to gonion.^10^ The Frankfort plane is a line that passes through the inferior borders of the orbital rim (orbitale) and the upper margin of the auditory meatus (porion) and is used to determine the NLA. Several studies describe the ideal NLA as 95° to 100° in men and 100° to 110° in women^9^^,^^14^^-^19 In addition, some have advocated slight variations in these parameters for patients of varying ethnic backgrounds.^15^^,^^16^^,^^20^^-^24 Aesthetic ideals have also been established for the position of nasion and the nasal radix.^25^^-^27

Most surgeons use the NLA as a parameter for measuring and adjusting tip position. However, other metrics such as the columellar facial angle and the nostril axis have also been used for quantifying tip rotation.^14^^-^16^,^^28^ Care must be taken to not confuse the columellar-labial angle with the NLA. The columellar-labial angle is the angle between the columella and the upper lip. Using this angle can lead to a false sense of the true tip position in cases where there is a pronounced caudal septum or nasal spine.^9^

### Techniques for rotation

The rhinoplasty surgeon faces tremendous variation not only in patient anatomy but also in desired patient outcomes. Not all patients require or desire the same amount of tip rotation. In addition, not all patients possess the adequate anatomical elements to employ solely one technique to alter tip position. After analyzing the entire nose and face and understanding the patient's wishes, the rhinoplasty surgeon can then employ the appropriate technique(s) to adjust the nasal tip.

Basic strategies focus on removing impediments to movement and rotation and stabilizing the cartilages once in the desired position.^9^ Some techniques used to correct the nasal tip include cephalic alar trim, suture reshaping and repositioning of the cartilages, vertical transection of the medial and lateral crura, resection of the caudal septum, insertion of tip grafts and struts, and insertion of alar strut and spreader grafts.^2^ Previous studies have outlined algorithmic approaches to delineating techniques to alter and define the nasal tip.^29^ The rhinoplasty surgeon typically selects from one or a combination of these techniques to achieve an aesthetically pleasing outcome.

Our study focuses on essentially one strategy to elevate the nasal tip—cephalic alar trim. While many techniques exist and are commonly used in combinations, we elected to observe how much the nasal tip would rotate purely by cephalic alar trim. As is the case for any surgical therapy, preoperative planning is essential to good outcomes. The observations from this study can help determine preoperatively whether other strategies need to be employed to achieve the desired result.

In performing the fresh tissue dissections, we used the closed cartilage delivery technique whereby marginal and intercartilagenous incisions are made to deliver the lateral cartilages. While the criticism of this technique has been that it distorts the cartilages, it does, however, allow for direct exposure and precision of resection.^2^^,^^14^ The open technique provides the best exposure to all the nasal elements; however, it potentially disrupts them as well.^30^ In addition, it has been suggested and demonstrated in previous anatomic studies that open techniques may result in a decreased nasal tip projection.^31^ As we intended to determine the effects of the change in one element, we adopted a technique that would best isolate manipulations to the alar cartilages.

Our aims were to determine the degree to which cephalic alar trim affects tip rotation. The clinical endpoint to this would be to aid surgeons in predicting how much the tip of the nose will elevate with a specific cartilage resection and thereby help plan their operation.

This type of cadaveric study has some intrinsic drawbacks. Although we used fresh cadavers from the University of Louisville Fresh Tissue Lab, the skin and soft tissues lack the elasticity and wound-healing capabilities of live tissue. These properties are inherent to rhinoplasty and likely to affect tip position. It was for this reason that we also employed a closed technique and did not close the incisions so as to disrupt as few elements as possible. In addition, it does not seem possible that such a study could be undertaken with live patients. While similar techniques are employed in the ethnic nose job, the degree to which the angles will change may be different with different ethnicities. Similarly, the cleft nose may respond differently. These drawbacks have been described in previous anatomical studies pertaining to rhinoplasty.^31^ (However, these results are not inconsistent with the practical experience of the senior authors, Wilhelmi and Florman.)

## CONCLUSION

Remodeling of the nose can be one of the most challenging components of facial plastic surgery. Typically, it is taught and performed in a seemingly artistic manner without an empiric base. With all the elements of the nose and intricacies of wound healing, the entire notion of rhinoplasty can be overwhelming. Most rely on surgeon experience to determine which technique to employ, or in our case, how much cartilage to resect in order to achieve a certain outcome. Our aim was to provide an element of predictability to less experienced and perhaps even seasoned rhinoplasty surgeons.

Knowledge of rhinoplasty anatomy and understanding of facial metrics is essential to planning and executing rhinoplasty precisely. Many techniques exist to rotate the nasal tip, with cephalic trim being one of the most popular.

The NLA is an essential aesthetic feature. Cephalic trim is a key maneuver in affecting the NLA. A 25% LLC cephalic trim correlates with an average change in the NLA of 6.47° ± 1.96°. We suggest that this study can aid in calculating and predicting the degree of tip elevation with a specified cephalic alar trim.

## Figures and Tables

**Table 1:** Data demonstrating the initial size of the LLC with respective NLAs*

Specimen	Gender	LLC width, mm	% Remaining	NLA, °	Incremental change, °	Total change, °
1	F	10	100	100		
			75	106	6	6
			50	113	7	13
			25	120	7	20
2	F	9.5	100	103		
			75	110	7	7
			50	117	7	14
			25	125	8	22
3	M	9.5	100	134		
			75	142	8	8
			50	146	4	12
			25	152	6	18
4	M	10	100	118		
			75	126	8	8
			50	132	6	14
			25	139	7	21
5	M	10.5	100	91		
			75	100	9	9
			50	104	4	13
			25	110	6	19
6	F	7	100	112		
			75	120	8	8
			50	125	5	13
			25	131	6	19
7	M	11	100	95		
			75	100	5	5
			50	105	5	10
			25	112	7	17
8	F	9.5	100	98		
			75	106	8	8
			50	114	6	14
			25	120	6	20
9	M	10.5	100	91		
			75	199	8	8
			50	103	6	14
			25	109	6	20
10	M	7	100	112		
			75	119	7	7
			50	124	5	12
			25	130	6	18
Average		9.45			6.47	19.6
SD		1.38			1.25	1.96

*Changes in NLAs with corresponding serial 25% resections of the lower lateral cartilages are also shown.

LLC indicates lower lateral cartilage; NLA, nasolabial angle, F, female; and M, male.

**Table 2:** Total and average changes in the nasolabial angle across all specimens with each resection

Specimen	Total change first resection	Total change second resection	Total change third resection
1	6	13	20
2	7	14	24
3	8	12	18
4	8	14	21
5	9	13	19
6	8	13	19
7	5	10	17
8	8	14	20
9	8	14	20
10	7	12	18
Average	7.4	12.9	19.6
